# A navigated, robot-driven laser craniotomy tool for frameless depth electrode implantation. An *in-vivo* recovery animal study

**DOI:** 10.3389/frobt.2024.1355409

**Published:** 2024-06-12

**Authors:** Fabian Winter, Patrick Pilz, Anne M. Kramer, Daniel Beer, Patrick Gono, Marta Morawska, Johannes Hainfellner, Sigrid Klotz, Matthias Tomschik, Ekaterina Pataraia, Gilbert Hangel, Christian Dorfer, Karl Roessler

**Affiliations:** ^1^ Department of Neurosurgery, Medical University of Vienna, Vienna, Austria; ^2^ Department of Medical Science Division, Medical University of Vienna, Vienna, Austria; ^3^ Advanced Osteotomy Tools, Basel, Switzerland; ^4^ Division of Neuropathology and Neurochemistry, Medical University of Vienna, Vienna, Austria; ^5^ Department of Neurology, Medical University of Vienna, Vienna, Austria; ^6^ Department of Biomedical Imaging, Medical University of Vienna, Vienna, Austria

**Keywords:** robotic, navigated, laser, craniotomy, epilepsy surgery, depth electrodes

## Abstract

**Objectives:** We recently introduced a frameless, navigated, robot-driven laser tool for depth electrode implantation as an alternative to frame-based procedures. This method has only been used in cadaver and non-recovery studies. This is the first study to test the robot-driven laser tool in an *in vivo* recovery animal study.

**Methods:** A preoperative computed tomography (CT) scan was conducted to plan trajectories in sheep specimens. Burr hole craniotomies were performed using a frameless, navigated, robot-driven laser tool. Depth electrodes were implanted after cut-through detection was confirmed. The electrodes were cut at the skin level postoperatively. Postoperative imaging was performed to verify accuracy. Histopathological analysis was performed on the bone, dura, and cortex samples.

**Results:** Fourteen depth electrodes were implanted in two sheep specimens. Anesthetic protocols did not show any intraoperative irregularities. One sheep was euthanized on the same day of the procedure while the other sheep remained alive for 1 week without neurological deficits. Postoperative MRI and CT showed no intracerebral bleeding, infarction, or unintended damage. The average bone thickness was 6.2 mm (range 4.1–8.0 mm). The angulation of the planned trajectories varied from 65.5° to 87.4°. The deviation of the entry point performed by the frameless laser beam ranged from 0.27 mm to 2.24 mm. The histopathological analysis did not reveal any damage associated with the laser beam.

**Conclusion:** The novel robot-driven laser craniotomy tool showed promising results in this first *in vivo* recovery study. These findings indicate that laser craniotomies can be performed safely and that cut-through detection is reliable.

## 1 Introduction

We recently introduced a navigated, robot-driven laser tool for implanting cranial depth electrodes as an alternative to frame-based procedures. This method has proven accurate and feasible in a cadaver study (Roessler et al., 2021). However, in the first *in vivo* trials, cut-through detection through its coaxial camera and optical coherence tomography (OCT) signals was obscured through inflowing liquids (Winter et al., 2021). Therefore, the cutting strategy was updated, and a new camera system was implemented to enhance cut-through detection (Winter et al., 2022). Previous trials were limited due to the non-recovery setting, and no postoperative imaging or histopathological analysis was conducted. Hence, no postoperative safety, histopathological analysis, or accuracy data is available. This study is the first *in vivo* recovery study using a robot-driven laser tool for craniotomies allowing postoperative neurological evaluation. In addition, postoperative imaging was performed to evaluate the entry point, target point, and angulation deviations.

## 2 Materials and methods

This study was approved by the local ethics committee and the Federal Ministry of Education, Science, and Research (#2020-0.468.726). This study was conducted per the European requirements (Directive EU/2010/63) and United States Food and Drug Administration Good Laboratory Practice regulations (21 CFR 58) and followed the test facility’s standards of care protocols. The surgeon performing the procedure was accredited with an EU Function A certificate for educational and training courses in laboratory animal sciences. The laboratory facility was specifically equipped, and the personnel was precisely trained to perform this magnitude of studies. The raw data supporting the conclusion of this study will be made available by the authors upon request.

### 2.1 Sheep specimens

Two sheep were used in this study. However, our sheep were kept at the facility to ensure a psychologically safe environment for those not tested. Therefore, no sheep were left alone in a cage at any time, which is crucial in animal care, especially in social animals such as sheep.

### 2.2 Preoperative planning

Each sheep underwent a head computed tomography scan using a Siemens Somatom Emotion 16 CT Scanner (Siemens, Munich, Germany). The scan parameters were set to a 0.75 mm axial resolution, with kernels H37s for tissue and H90s for bone. The voxel settings were SL 0.75/16 × 0.6/p 0.8; the imaging parameters were 130 kv and 240 mA. Digital imaging and communications in medicine data were processed and validated into STL files using surgical planning software (Neuro SEEGPlan software, Advanced Osteotomy Tools AG, Basel, Switzerland). The data were transferred to the graphical user interface of the laser osteotome (CARLO® primo +, Advanced Osteotomy Tools AG). Preoperative planning was performed to define the trajectories and entry points for the depth electrodes.

### 2.3 Registration procedure

**FIGURE 1:**
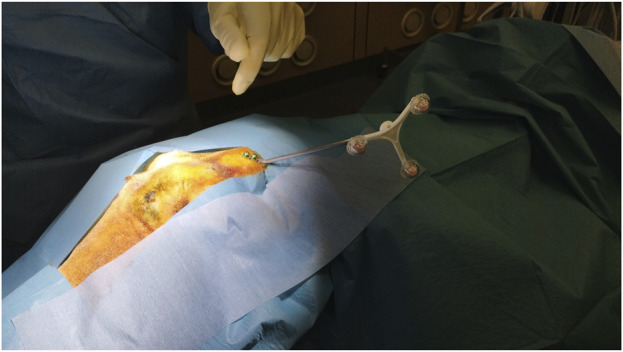
Implantation of patient marker for navigation reference after surgical prepping.

The laser head of the laser osteotome CARLO® primo+ was mounted on a 7-axis robotic arm (KUKA AG, Augsburg, Germany) and guided by an infrared camera navigation system that tracked two marker sets. One is integrated into the laser head, and the other is a patient marker attached to the posterior skull plate (Figure 1). Surgery was performed with the sheep specimen in the prone position, and a custom-made cushion stabilized the head. The position of the sheep head relative to the attached marker was determined by identifying four screws (Medartis AG, Basel, Switzerland) set as physical landmarks, which were implanted on the cranium before the preoperative CT scan for the registration procedure. A registration accuracy of <1.0 mm root mean square was considered acceptable. The registration procedure has already been described in our previous works (Winter et al., 2021; Winter et al., 2022).

### 2.4 Anesthesia

The sheep were sedated by intravenously administering midazolam (0.3 mg/kg) and ketamine (4 mg/kg). Anesthesia was then induced with intravenous propofol (2%, 5 mg/kg). Orotracheal intubation was performed for volume-controlled ventilation. Isoflurane 1%–2% and sevoflurane 3%–4% were used to maintain anesthesia throughout the procedure. Before imaging, ketamine (3 mg/kg) and detomidine (0.07 mg/kg) were used to calm the sheep and allow steady imaging.

### 2.5 Surgical procedure

Two linear paramedian skin incisions were made to display the cranium. One large spreader was inserted to prevent skin flaps from obscuring the operative field. As previously described, the laser osteotome uses a 2.94 μm erbium-doped yttrium aluminum garnet (Er:YAG) laser (Baek et al., 2015; Roessler et al., 2021; Winter et al., 2021). The cutting strategy was previously updated to facilitate cut-through detection (Winter et al., 2022). Once cut-through detection was confirmed on either the coaxial camera system or OCT signal, the anchor bolts were placed, and the depth electrodes were inserted. The dura was perforated using a standard monopolar probe (Roessler et al., 2016). The alignment of the anchor bolts was confirmed by aiming the laser head according to the planned trajectory.

### 2.6 Termination of animals

Animals were premedicated with 0.3 mg/kg midazolam intravenously and 4 mg/kg intravenous ketamine. Afterward, according to the termination protocols of the institute, the animals were euthanized using intravenous pentobarbital (300 mg/kg). This aligned with the ethical and official guidelines for good scientific practice and animal welfare.

### 2.7 Postoperative imaging

Postoperative MRI and CT scans were conducted to assess the accuracy of the planned entry points and trajectory angulations. This was achieved by integrating the electrodes, visible on CT, into the planning software. Based on the actual positions of the electrodes, new trajectories were established and compared with the preoperative plans within the software. We then quantified the deviations between the preoperatively planned trajectories and the postoperative trajectories. In addition, postoperative imaging was used to assess potential bone and cortical structure damage.

### 2.8 Histopathological analysis

After the termination of the animals, the brains were removed and fixed in formalin. Brain cutting was performed, and tissue samples were taken from the bone, dura, and brain surrounding the trajectory after removing the implanted electrodes. Tissue samples were routinely embedded in paraffin. Immunohistochemistry was performed using the DAKO Envision System kit (DAKO, Glostrup, Denmark) by applying an antibody against the glial fibrillary acidic protein (GFAP, polyclonal rabbit, 1:3000, DavoCytomation, Glostrup, Denmark). Diaminobenzidine was used as the chromogen.

### 2.9 Statistical analysis

Descriptive analysis was performed for all bone channels and entry points. The pre-operative planned trajectories were compared to the post-operative imaging. The deviation was calculated as the Euclidean distance, defined as the square root of the sum of the squares of the differences between the X,Y,Z coordinates of the planned versus effective trajectories.

## 3 Results

A total of 14 depth electrodes were inserted into the two sheep specimens. Both anesthetic protocols showed no irregularities, and both sheep were extubated within minutes of the procedure. Setscrews, inserted prior to the CT scan, served as reference points for landmark identification. The registration process was completed within 3 min, achieving a root mean square error of 0.6 mm. One sheep was euthanized on the same day and a postoperative MRI was performed which did not reveal any hemorrhage, infarction, or other brain damages. Postoperative CT scans of both specimens 1 week after the procedure also did not show any signs of unintended damage and was used to verify accuracy of the electrodes.

### 3.1 Surgical procedure–precision laser trephines

After surgical prepping and two linear paramedian skin incisions, a straight spreader displayed the surgical field. After automatic laser ablation, until the set thickness of the remaining bone was reached, based on comparison of the CT data and the OCT read-out, the procedure was switched to a manual mode in which the surgeon controlled further ablation until cut-through was detected. The median ablation time for each burr hole was <3 min.

### 3.2 Accuracy of entry points

**FIGURE 2:**
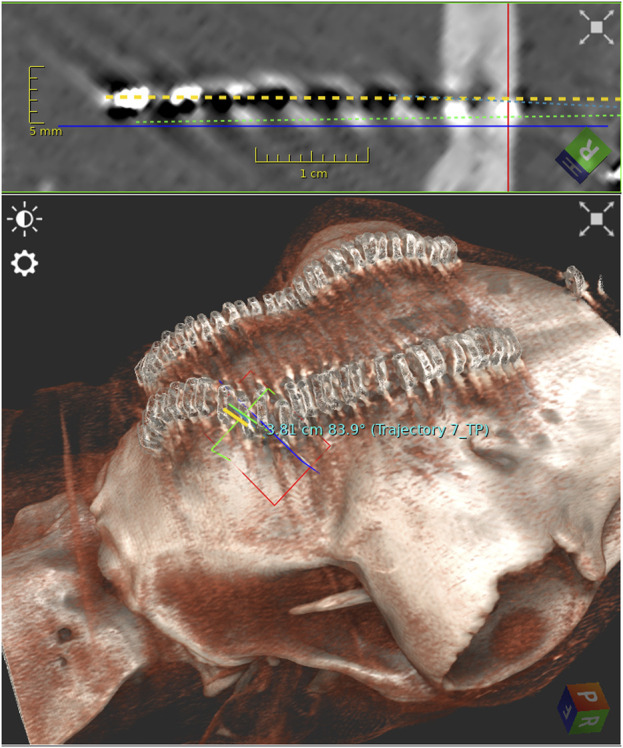
Postoperative computed tomography imaging with 3-dimensional reconstruction showing the planned trajectory and depth electrode alignment.

**FIGURE 3:**
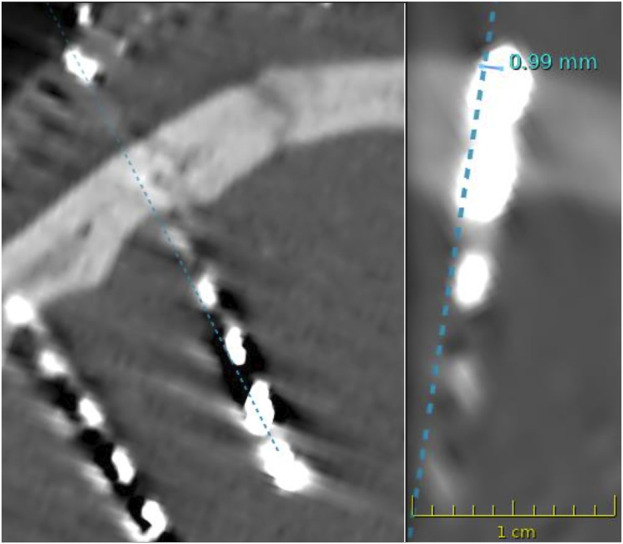
Postoperative computed tomography imaging showing entry point deviations of < 1 mm.

The registration accuracy was <1.0 mm for both specimens. The average bone thickness was 6.2 mm (range 4.1 mm–8.0 mm). The average angulation was 79.8° (range 65.5°–87.4°) (Figure 2). Entry point deviation ranged from 0.27 mm to 2.24 mm (average 1.51 mm) (Figure 3). The accuracy of target points varied between 0.00 mm and 9.26 mm, with an average of 3.06 mm (Table 1).

**TABLE 1:** Properties of all planned trajectories.

Trajectory	Bone thickness	Angulation	Deviation of entry point	Deviation of target point
1	6.8	69.7	0.98	1.92
2	6.1	65.5	0.81	1.70
3	6.8	79.7	1.16	3.22
4	4.1	78.3	0.61	4.87
5	5.7	79.5	0.27	1.39
6	8.0	84.5	2.24	3.33
7	6.6	83.4	1.97	2.69
8	7.3	82.7	2.01	2.03
9	4.5	87.4	2.03	9.26
10	5.4	86.1	1.89	4.03
11	5.9	82.6	1.67	4.10
12	5.6	69.7	1.89	0.00
13	7.0	83.7	1.81	1.23
14	6.6	84.1	1.76	3.00

All values in mm, besides angulation in degrees.

### 3.3 Insertion of depth electrodes

**FIGURE 4:**
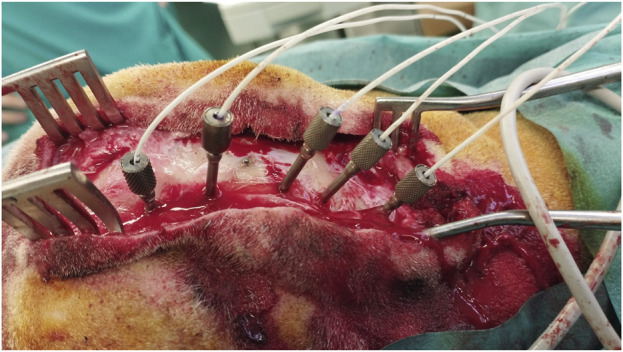
Multiple depth electrodes with different angulations implanted through anchor bolts after laser ablation.

All the planned laser trephines were created. Cut-through detection using a coaxial camera and OCT signals was feasible. Insertion of anchor bolts was possible without complications, independent of bone thickness and angulation (Figure 4). Haptic feedback confirmed a tight fit and was experienced in all cases. Depth electrodes were unpretentiously implemented in all the burr holes. In four burr holes, monopolar coagulation was performed to stop minor bleeding through the anchor bolts before inserting the depth electrode. This technique has been previously described (Winter et al., 2022).

### 3.4 End of procedure–skin closure

**FIGURE 5:**
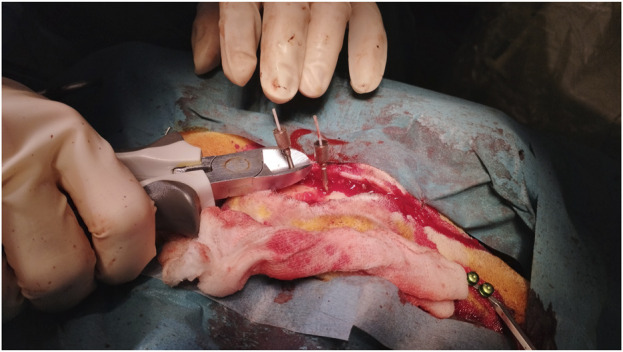
Cutting depth electrodes and anchor bolts allowing skin closure to prevent postoperative manipulation and reduce infarction risk.

Electrodes and anchor bolts were cut at the bone surface level to prevent postoperative manipulation and risk of infarction (Figure 5). As sheep are known for their curiosity and increased stress levels if something seems wrong, we wanted to ensure no postoperative attempts to remove protruding foreign objects. After subcutaneous stitches, the skin was closed with skin clips.

### 3.5 Histopathological analysis

**FIGURE 6:**
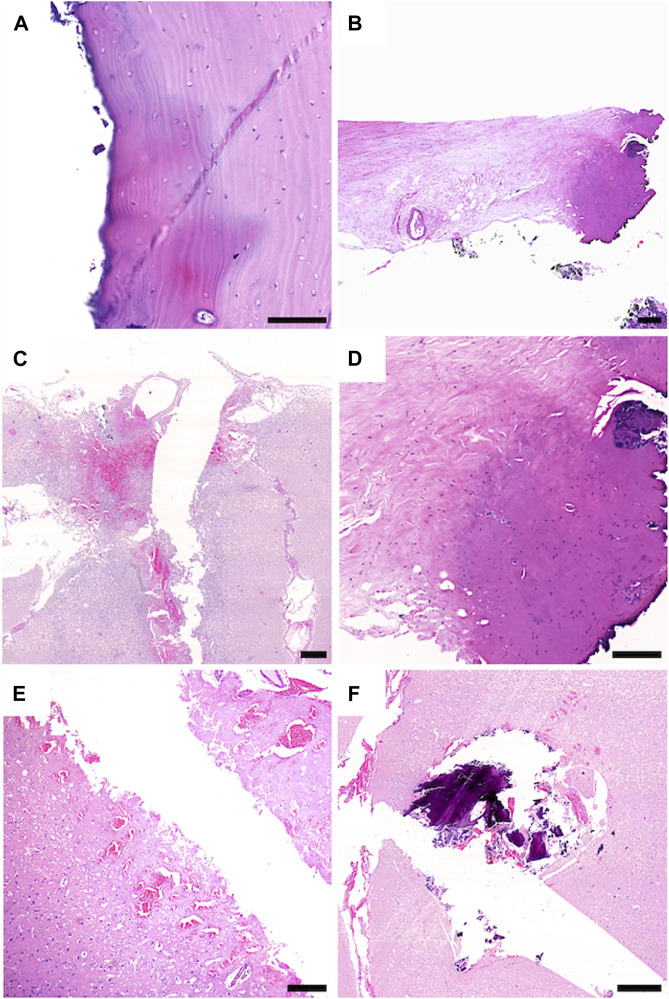
Sheep 2: **(A–D)**, Sheep 1: **(E,F)**; **(A)** The stitch channel of the bone is sharply delineated and shows no connective scar tissue formation. The bone tissue surfacing the stitch channel shows a thin area with thermocoagulation artifacts (dark colored area). **(B,D)** The dura tissue surfacing the stitch channel shows a limited field with thermocoagulation artifact (dark colored area). **(C)** CNS tissue with tissue defect, whit limited surrounding tissue damage. **(F)** CNS tissue surrounding the stich channel shows fresh hemorrhages and edema. **(E)** within the stitch channel, tiny fragments of bone and fresh hemorrhage are visible. Scale Bars: 100 μm: **(A)**, 150 μm: **(D)**, 200 μm: **(B)**, 500 μm: **(C–F)**.

Tissue samples of the bone, dura, and cortex were retrieved to analyze the tissue around the ablation areas and trajectory sides. Laser-generated ablation penetrated the bone, dura, and underlying brain tissue. All tissue samples at the site of laser craniotomy showed minimal thermocoagulation artifacts with little tissue reaction. In the bone and dura, thin areas of thermocoagulation artifacts surfacing the laser-induced ablation sides were noted. While thin areas of thermocoagulation artifacts were observed on the surfaces of the laser-induced ablation sites in the bone and dura, the underlying brain tissue exhibited no signs of thermocoagulation. Instead, nonspecific changes were noted around the stitch-channel, including signs of edema and minor fresh bleedings. Importantly, there were no significant reactive tissue changes or scar formations in the brain tissue (Figure 6).

## 4 Discussion

This is the first study reporting data on robotic laser cranial burr holes for frameless implantation of depth electrodes in an *in vivo* recovery animal study. Notably, numerous reports exist on implementing lasers in medicine, especially in cutting bone (Baek et al., 2015; Augello et al., 2018). The benefits of laser osteotomies are high precision and freedom of geometry (Winter et al., 2022). So far, combining a laser osteotome with a reliable navigation system has been a challenge without losing the geometry within the operating room. The laser osteotome setup used in this study has already been certified in oral and maxillofacial surgery (Baek et al., 2015). It uses an Er:YAG laser ablating bone while heat to the surrounding tissue is minimal due to a persistent cooling spray during ablation (Stübinger et al., 2009). However, laser ablation has prolonged operating times due to relatively low cutting speed. In this series, the median time for ablating burr holes was <3 min. This allows implantation times of <12 min per electrode, below the previously published data for other frameless robotic devices (Dorfer et al., 2014).

The laser osteotome has already been tested and approved for human trials in oral maxillofacial surgery (Baek et al., 2015). After performing the first cranial trials in cadavers and *in vivo* non-recovery studies, this is the first study to prove that the workflow is safe in an *in vivo* recovery setting (Roessler et al., 2021; Winter et al., 2021; Winter et al., 2022). Anesthetic protocols showed no irregularities during surgery in both specimens. One specimen was euthanized on the day of the procedure while the other was euthanized a week later. Posttermination MRI and CT scans showed no signs of hemorrhage, infarction, or other cortical damage. However, histopathological analysis revealed generalized edema in one sheep, most likely due to prolonged narcosis. Small laser trephines were ruled out as potential morbidities of general edema by the involved neuropathologists. There were no signs of neurological impairments in the sheep that was kept alive for 1 week after the procedure, and the specimen could return to the other sheep with no signs of behavioral abnormalities. Postoperative imaging showed no signs of hemorrhage, infarction, or other pathologies, similar to the first specimen. The histopathological analysis did not reveal any significant tissue abnormalities. The bone and dura showed the expected tissue reaction and scar formation without unexpected damage. This supports safe instrumentation with a laser osteotome.

Accuracy is the key to successful depth electrode implantation. Deviations in the entry point or angulation can lead to injury of blood vessels or failure to localize seizure onset zones (Dorfer et al., 2014; Mullin et al., 2016; Roessler et al., 2016; Dorfer et al., 2017; Lu et al., 2021). Frameless techniques are comparable to frame-based methods regarding morbidity and accuracy (Cardinale et al., 2013; Dorfer et al., 2014; González-Martínez et al., 2016; Hou et al., 2016). However, the craniotomy direction, especially the angulation, are more flexible and less reliable in frameless techniques (Lu et al., 2021). This novel robotic-assisted frameless laser ablation method showed entry-point accuracy results comparable to already published frame-based and frameless methods (Dorfer et al., 2017; Lu et al., 2021). However, this report does not differentiate between the choice of imaging for referencing and the referencing method itself, which has also been reported to influence accuracy (Vakharia et al., 2017). Target point accuracy varied between 0.00 mm and 9.26 mm in this study. This range could result from gross metal cutting of screws and bolts before skin closure. Also, incorrect electrode depth measurement and insertion may have resulted in deviated target points as the one electrode with the highest deviation of more than 9 mm had contact with the skull base and drifted off alongside the skull base bone. However, this was not attributed to the frameless laser osteotome.

An often-reported challenge for frameless techniques is high skew-angle trajectories with larger errors in accuracy due to drill sliding at the entrance of the skull (Iordanou et al., 2019; Lu et al., 2021). As previously reported, drill sliding at the skull and high skew-angle trajectories are not feasible with laser ablation (Winter et al., 2022). To reduce the risk of drill sliding, slight pressure is recommended when drilling is initiated (Iordanou et al., 2019; Lu et al., 2021). However, this can lead to uncontrolled fractures in thin bone segments, which may prohibit screw placement. The bone thickness of performed burr holes in this study varied from 4.1–8.0 mm, indicating that even thin bone segments can be laser ablated precisely without mechanical stress fractures. Screw implantation was feasible in all burr holes, allowing safe implantation of depth electrodes.

**FIGURE 7:**
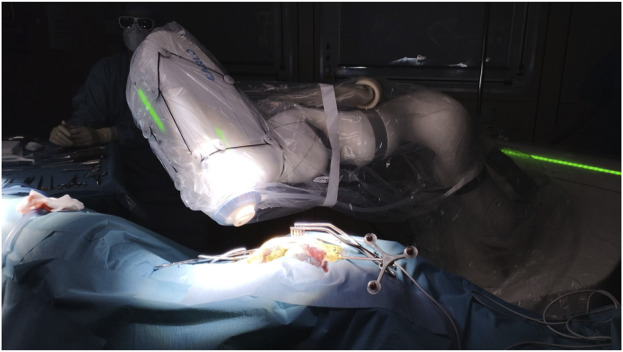
Operating room set-up during laser ablation. Robotic-assisted frameless laser osteotome in action.

As previously described, this robotic laser osteotome setup allows in-room interactions between the surgeon and the device (Roessler et al., 2021; Winter et al., 2021). The in-room memory function of the robotic arm aided this. The surgeon can easily access the surgical field when the robotic arm is retracted. Without further ado, the robotic arm finds itself in the same position (Figure 7). This ensures the cooperation of an external device and the surgeon in the already limited space around the operating table.

Laser osteotomies result in sharp cutting edges. In postoperative histopathological analysis, a demarcation zone in which the homogenous lamellar bone matrix structure was changed to a diffuse fibrous-like structure was approximately 2 mm. This is similar to previously published data on Er:YAG laser application in maxilla surgery (Stübinger et al., 2009). Osteocyte lacunae directly adjacent to the ablation site contained osteocytes with normal structural characteristics.

## 5 Limitations

The number of burr holes performed was limited to only two animal specimens. However, considering the “do not harm” ethical aspects in animal trials, we believe in having accounted for sufficient safety and feasibility data with 14/14 successful laser-ablated burr holes.

## 6 Conclusion

The frameless robot-driven laser tool showed promising results in the first *in vivo* recovery study for depth electrode implantation. This study demonstrates the feasibility and validity of cut-through detection after laser ablation. These results can be the basis for the first-in-human trials of frameless laser ablation craniotomies.

## Data Availability

The raw data supporting the conclusion of this article will be made available by the authors, without undue reservation.
